# When Music and Long-Term Memory Interact: Effects of Musical Expertise on Functional and Structural Plasticity in the Hippocampus

**DOI:** 10.1371/journal.pone.0013225

**Published:** 2010-10-05

**Authors:** Mathilde Groussard, Renaud La Joie, Géraldine Rauchs, Brigitte Landeau, Gaël Chételat, Fausto Viader, Béatrice Desgranges, Francis Eustache, Hervé Platel

**Affiliations:** 1 Inserm-EPHE-Université de Caen/Basse-Normandie, Unité U923, GIP Cyceron, CHU Côte de Nacre, Caen, France; 2 Département de Neurologie, CHU Côte de Nacre, Caen, France; University of Groningen, Netherlands

## Abstract

The development of musical skills by musicians results in specific structural and functional modifications in the brain. Surprisingly, no functional magnetic resonance imaging (fMRI) study has investigated the impact of musical training on brain function during long-term memory retrieval, a faculty particularly important in music. Thus, using fMRI, we examined for the first time this process during a musical familiarity task (i.e., semantic memory for music). Musical expertise induced supplementary activations in the hippocampus, medial frontal gyrus, and superior temporal areas on both sides, suggesting a constant interaction between episodic and semantic memory during this task in musicians. In addition, a voxel-based morphometry (VBM) investigation was performed within these areas and revealed that gray matter density of the hippocampus was higher in musicians than in nonmusicians. Our data indicate that musical expertise critically modifies long-term memory processes and induces structural and functional plasticity in the hippocampus.

## Introduction

Becoming a skilled musician requires a lot of practice and this kind of learning relies on multiple faculties (e.g. perception, memory and motor abilities) [1]. The skills developed by musicians induce specific connections and interactions between various brain areas [2]. Previous structural and functional studies have demonstrated the effects of musical training on the brain [1], [3], but fMRI investigations are scarce and have mainly focused on auditory, motor or somatosensory tasks (see for examples [4]–[8]). The functional changes associated with musical training, in these fMRI studies, take the form of stronger activation, mainly in the temporal cortex (particularly the middle temporal gyrus) and somatosensory areas [4]–[8]. Thus, the impact of musical training on brain activity has been explored through various experimental paradigms, but has never been assessed with fMRI using a task that specifically focuses on long-term musical memory retrieval. In nonmusicians, this kind of task activates a complex bilateral neural network including in all studies the inferior frontal and superior temporal gyri [9]–[12] predominantly in the left hemisphere as highlighted in our previous researches.

Moreover, repeated practice, such as musical training, is known to induce structural differences in the brain [13]. In fact, the main differences observed consist of the enlargement of several brain regions in musicians [3]. The anteromedial portion of Heschl's gyri [14]–[16], the corpus callosum [6], [17], and the planum temporale [18], [19] are all larger in musicians than they are in nonmusicians. In addition, musical experience during childhood appears to have an influence on structural development, especially in the auditory and motor cortices [20]. This structural plasticity induced by musical practice is also supported by changes in white matter, with corticospinal tract modifications [21].

Following functional neuroimaging works concerning neural substrates of long-term musical memory [9], [22], the aim of the present study was to explore the impact of musical expertise on both functional and structural modifications of the brain. For this purpose, we selected 20 young musicians who had been playing music without interruption until the time of the study (number of years of practice: 15.3±3.67) and 20 young common listeners but strictly nonmusicians (Table 1). They were scanned using fMRI during a musical semantic memory task in which they had to rate the familiarity of 60 melodies (purely instrumental tonal excerpts, see supplemental data for the list of musical excerpts) on a 4-point scale (from “non familiar” = 1 to “extremely familiar” = 4). This task required subjects to compare the music that was played to them with melodies stored in their semantic memory. Thus, to identify the brain regions associated with musical expertise during this familiarity task, we analyzed the effects of familiarity on cerebral activity based on the subject's response for each melody.

**Table 1:** Population characteristics.

	Nonmusician group	Musician group
*Number*	20	20
*Women/men*	10/10	10/10
*Age: mean ± SD*	24.55±3.80	22.85±3.05
*Educational background* £ *^,^* * *: mean ± SD*	16.35±2.03	15.15±0.99

*SD  =  standard deviation*.

£ *number of years of schooling*.

*significant difference at p<0.05.

Finally, consistent with the idea of training-induced structural reorganization in the adult brain [13], we then conducted a voxel-based morphometry (VBM) investigation, taking exclusively into account the areas pinpointed when comparing the fMRI data of musicians and nonmusicians, to examine the influence of the musical expertise on structural plasticity.

## Results

### Demographic data

The mean education level differed significantly between musicians (15.15±0.99) and nonmusicians (16.35±2.03), t(38) = 2.37, p<0.05, and no significant difference was observed for age.

### Behavioral data

**Figure 1:**
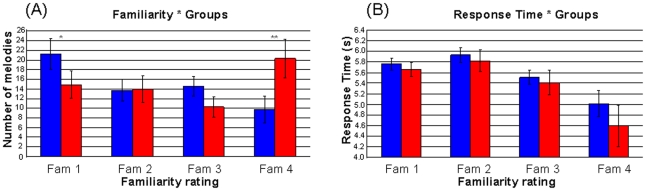
Behavioral results. Interaction effects between (A) familiarity ratings (responses numbers) and groups (musicians and nonmusicians), and (B) response time (in seconds) and groups (musicians and nonmusicians). Significant differences at p < .05 (*) and p < .001 (**).

A two-way analysis of variance (ANOVA) was performed with the group as between-subjects factor with two modalities (nonmusicians and musicians), and the familiarity rating as a within-subject factor with four levels (Fam1, Fam2, Fam3, and Fam4). The ANOVA revealed (1) no main effect of group, *F*(1, 38) = 0.07, *p* = 0.79, but (2) a main effect of familiarity, *F*(3, 114) = 4.30, *p*<0.006, indicating a highest rating for totally unfamiliar (Fam1; mean value  = 18.05) and extremely familiar (Fam4; mean value  = 15.02) musical excerpts and lowest rating for Fam2 (mean value  = 13.82) and Fam3 (mean value  = 12.37) levels, and (3) a significant interaction effect between the group and familiarity ratings, *F*(3, 114) = 10.43, *p*<0.001. On the basis of this significant interaction, *post hoc* comparisons (Tukey's HSD) were performed in order to identify specific effects (Figure 1A). In the Fam1 and Fam4 conditions, we observed a significant difference (*p*<0.05, *p*<0.001) in the number of melodies reported as unfamiliar between nonmusicians and musicians. As expected, nonmusicians judged more melodies to be totally unfamiliar (i.e. Fam1) and musicians judged more excerpts to be extremely familiar (i.e. Fam4).

The ANOVA conducted on response times revealed (Figure 1B) (1) no main effect of group, F(1, 38) = 2.10, p = 0.1, (2) a main effect of familiarity ratings, F(3, 114) = 81.44, p<0.001, indicating the more familiar the melodies were, the shorter the response times and (3) no interaction effect between the group and familiarity ratings, F(3, 114) = 2.25, p<0.08.

Following the scanning session, a debriefing session was proposed to determine whether the melodies had evoked personal memories or mental imagery. This debriefing revealed that the musical excerpts evoked personal memories in 85% of musicians, but in only 30% of nonmusicians. When musicians reported having personal memories (e.g. “I've played this melody before”; “I learnt this excerpt during my formal musical training”), most of them also had associated mental imagery (e.g. “I can see myself playing this melody”), which was not the case for nonmusicians.

### Imaging data

#### Effect of familiarity

**Figure 2:**
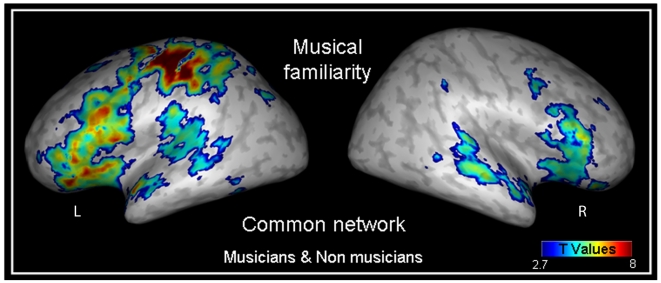
Common network between groups for the musical familiarity task. This network was composed of a left extensive cluster including motor, and inferior frontal areas, the bilateral superior temporal gyri, the right cerebellum and the inferior frontal gyrus and the left inferior parietal gyrus (see also Table 2). Contrasts are displayed at p<0.005 (uncorrected) and superimposed onto an inflated reconstruction of an MNI template brain using Anatomist software (www.brainvisa.info). Abbreviations: L = left hemisphere, R = right hemisphere.

A conjunction analysis revealed that, in both musicians and nonmusicians, familiarity increased activity in an extended network including left motor areas (attributed to the right-hand responses for the familiar excerpts), the bilateral inferior frontal and superior temporal gyri (significantly more prominent in the left hemisphere at *p*<0.001, t(39) = 8.13 and t(39) = 9.79, respectively), the right cerebellum and the left inferior parietal gyrus (Figure 2 and Table 2).

**Table 2:** Functional activations.

Anatomical location	Cluster size (in voxels)	x	y	z	Z score
***Conjunction Musical familiarity***					
Left inferior frontal gyri (including motor areas)	20344	−46	−26	58	>7.80***
		−44	30	4	
Right cerebellum	2024	18	−52	−22	6.64***
Right inferior frontal gyrus	1408	34	32	−10	4.70***
Bilateral superior temporal gyri	780	−52	−18	22	4.42***
	317	54	−26	−4	4.34***
Left inferior parietal gyrus	115	−32	−66	44	3.76
***Musicians vs. Nonmusicians***					
Bilateral hippocampus (anterior part)	439	−12	−14	−14	4.53***
	476	40	−26	−18	4.24***
Bilateral calcarine (including occipital and lingual gyri)	1730	20	90	−8	4.14***
		−10	−52	2	3.96***
Bilateral orbitomedial frontal gyri	276	−8	58	−10	4.06**
		8	62	−6	3.57**
Middle cingulate cortex	187	0	−22	56	3.79*
Bilateral superior temporal areas (including Heschl's gyri)	73	−56	−6	10	3.67
	121	42	−32	14	3.67
Left Cerebellum	67	−12	−38	−12	3.64

***whole brain cluster corrected at 0.001.

**whole brain cluster corrected at 0.002.

*whole brain cluster corrected at 0.02.

Brain activation obtained in the conjunction musical familiarity analysis and brain areas in which activity was positively correlated with the musical familiarity more so in the musicians than in the nonmusicians group.

Coordinates x, y, z (mm) are given in standard stereotactic MNI space. All regions listed are statistically significant at the p<0.001 uncorrected level. We reported also the whole-brain cluster correction threshold at *** p<0.001, ** p<0.002 and *p<0.02.

**Figure 3:**
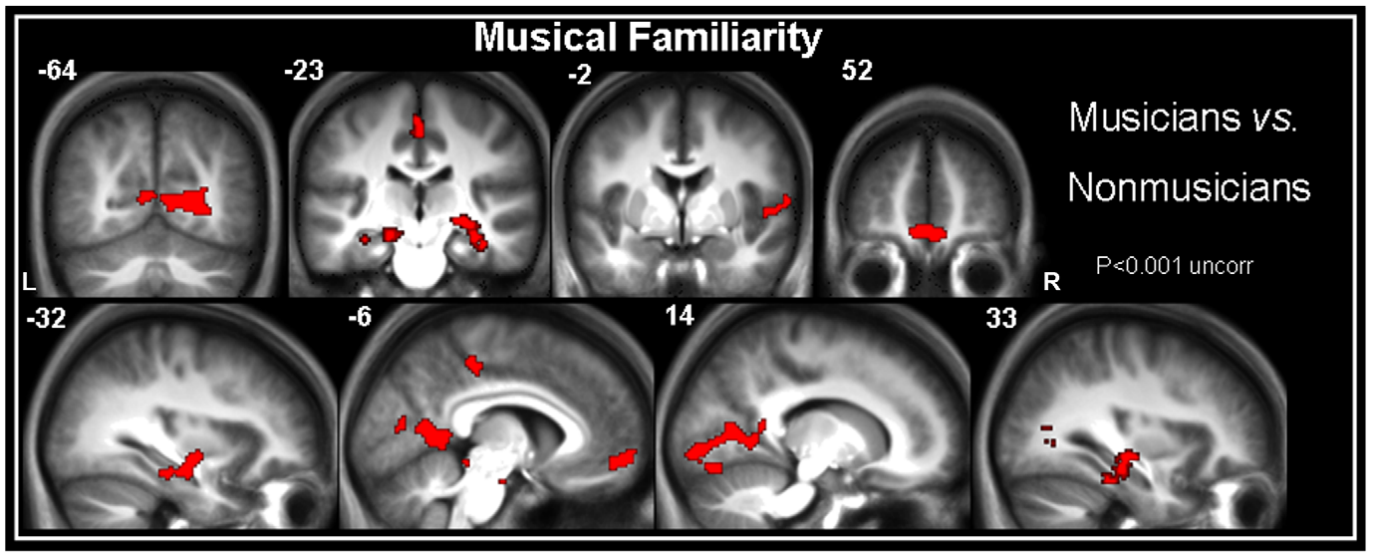
Higher activations in musicians vs. nonmusicians during the musical familiarity task. Areas of significantly stronger activation in musicians, compared with nonmusicians, during the musical familiarity task (see also Table 2). The bilateral anterior portion of the hippocampus (extending to the entorhinal cortex on the left and into the parahippocampus on the right), the bilateral calcarine (extending to the occipital and lingual gyri) and orbitomedial frontal gyri, the middle cingulate cortex and the bilateral superior temporal areas, including Heschl's gyri, were associated with increased familiarity for the musicians. Contrasts are displayed at p< 0.001 (uncorrected) and superimposed onto the average of normalized brains using MRIcron software. Abbreviations: L = left hemisphere, R = right hemisphere.

To highlight the effect of musical training, we focused on those areas where the familiarity-related activity increased more for musicians than for nonmusicians. The comparison between musicians and nonmusicians revealed greater activity in the bilateral (significantly more prominent in the left hemisphere at *p*<0.001, paired t-test) anterior portion of the hippocampus (extending into the entorhinal cortex on the left, and into the parahippocampal gyrus on the right), both sides of the calcarine and orbitomedial frontal gyri, and the middle cingulate cortex and bilateral superior temporal areas, including Heschl's gyri (Figure 3 and Table 2). We found a similar pattern of activation when we controlled for differences in response time, including this parameter as a covariate (data not shown).

No brain area exhibited changes in activity with decreasing familiarity when comparing musicians with nonmusicians. Similarly, the comparison of nonmusicians to musicians failed to reveal any area in which activity varied with familiarity.

#### Voxel-based morphometry analyses

**Figure 4:**
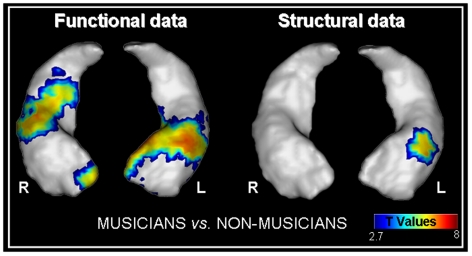
Functional and structural differences between musicians and nonmusicians. Contrasts are displayed at p<0.005 (uncorrected) and rendered onto a hippocampus mesh using Anatomist software (www.brainvisa.info). Abbreviations: L = left hemisphere, R = right hemisphere.

A morphometric analysis focusing on those areas that had previously been found to be significantly activated in the fMRI data analysis (comparison between musicians and nonmusicians, Figure 3) revealed higher gray matter density for musicians compared with nonmusicians, but only in the left hippocampal head ([−32; −12; −16], Figure 4). The effect size (Cohen's *d*; [23]) in the hippocampal voxel of highest statistical significance was large (*d* = 1.15). However, this comparison was also performed at a whole-brain level and the main structural difference was observed in the left hippocampus, reinforcing our result (data not shown).

#### Voxel-based multimodal analyses

Regarding the functional and structural brain differences observed in the hippocampus of musicians compared with nonmusicians, we wanted to ensure that the stronger activation of this area in musicians did not merely reflect the structural difference between the two groups.

After controlling for structural changes between musicians and nonmusicians with Biological Parametric Mapping toolbox (BPM; [24]) implemented in SPM5 we found the same levels of activation as in the previous fMRI analyses. This result confirms that the hippocampus is more strongly involved in musical familiarity judgement tasks in musicians than it is in nonmusicians, regardless of gray matter density differences between the two groups.

## Discussion

In line with previous neuroimaging studies [9]–[12], [22], our fMRI data revealed activation of the neural network known to be involved in musical familiarity tasks in both musicians and nonmusicians. This network encompassed the bilateral inferior frontal and superior temporal gyri (more extended in the left hemisphere), the right cerebellum and the left inferior parietal gyrus.

The originality of our study relies in the fact that we investigated, for the first time, the impact of musical expertise on brain plasticity during a musical long-term memory task. Our main result indicates that musicians exhibit both greater activity and gray matter density in the anterior part of the left hippocampus.

### Familiarity or recollection?

The musical familiarity task required participants to compare the melodies they heard with those already stored in their semantic memory (they knew that they knew a particular melody [25]). Recognition can be based on two distinct processes named familiarity and recollection. Recollection is defined as the retrieval of contextual details and includes sensory information that was experienced during the initial encounter, whereas familiarity corresponds to a mental awareness of a prior experience in the past, in the absence of mental reinstatement as in recollection (for review [26]).

The bilateral hippocampal activation observed with increased musical familiarity in musicians raises the question of which of familiarity or recollection processes are most involved in this task, a key issue in the familiarity/recollection debate. Indeed, although the hippocampus and other parts of the medial temporal lobe are known to contribute to recollection [26]–[28], for some authors these areas appear also to subserve familiarity processes [29]–[32].

In the present study, participants had only to judge the level of familiarity of 60 excerpts of melodies on a 4-point scale (from totally unfamiliar to extremely familiar). This required to search in their semantic memory if they had already stored the melodies. During this task, musicians gave significantly more excerpts the maximum rating (“I know this melody perfectly”) than nonmusicians did. In the course of their musical life, musicians obviously have the opportunity to store more melodies in their semantic memory than nonmusicians. The postexperiment debriefing demonstrated that melodies judged to be “extremely familiar” evoked personal memories for 85% of musicians, as opposed to just 30% of nonmusicians. As reported for familiar faces [33], melodies rated as familiar by musicians formed part of their semantic knowledge, but had extensive associations with contextual and episodic (phenomenological) components. When musicians hear a familiar melody, they may re-experience personal memories (e.g. melody played in a past concert) but, due to many hours of practicing, it is unlikely that each melody was systematically associated with a specific (or unique) event complete with all its spatiotemporal details. Thus, in our study, the left hippocampus may rather subserve a subjective recollection than a truly objective process, with specific contextual details, consistent with Spaniol et al.'s [34] proposal. The comparison between musicians and nonmusicians also revealed higher activity in the visual primary cortex, orbitomedial frontal gyri, middle cingulate cortex and bilateral superior temporal areas, including Heschl's gyri. These regions are known to be involved in autobiographical memory, characterized by vividness of details and self-referential processing [35], [36]. Activation of the visual primary cortex indicates that familiar melodies were richer in vivid sensory details and thus in visuospatial imagery [37] for musicians than for nonmusicians, due to the former's musical experience. Musical expertise may enhance mental imagery when listening to a familiar tune (participants had their eyes closed during the task) [38], [39]. The medial frontal cortex activation reinforces this view, indicating that when musicians recognized melodies they had played before, they engaged in self-referential processing [36], [40] associated with visuospatial imagery and mental re-experiencing. Moreover, the medial frontal cortex is also known to be involved in memory retrieval [41], supporting the hypothesis of a more detailed retrieval for musicians. Finally, the bilateral activation in the temporal areas, including Heschl's gyri, reflects musical imagery processes [42] that occurred while musicians listened to the melodies.

To sum up, the pattern of brain activation observed in musicians compared to nonmusicians reflects a constant interaction between episodic and semantic memory during the familiarity task as suggested, for example, by the MNESIS model [43]. This pattern underlies the involvement of subjective recollection associated with visuospatial imagery, mental re-experiencing and self-referential processes (see [44], for a cognitive neuroscience conception of autobiographical memory) during this task.

### Training-dependent plasticity

The influence of intensive training on cognitive function and brain structure has already been demonstrated in several fields of expertise (such as chess [45], mathematics [46], taxi-driving [47] and juggling [48]). To test the hypothesis that repeated practice, such as musical training, also induces structural changes, we performed a VBM investigation, focusing on the areas that had been activated in musicians versus nonmusicians during fMRI analyses. Musicians had significantly higher gray matter density in the head of the left hippocampus. In their famous London taxi drivers study, Maguire and colleagues [47] found that the posterior part of the hippocampus was modulated by spatial expertise. Regarding the head of the hippocampus, few hypotheses have been put forward. Although the HIPER model [49] proposed that the anterior part of the hippocampus is dedicated to encoding processes, the anatomical hippocampal specialization has yet to be confirmed. Two studies have indicated a correlation between the gray matter density of the head of the hippocampus and performance on a verbal memory test (immediate recall) [50], [51]. Our results extend these findings to long-term musical memory retrieval. Musical skills developed by musicians induce specific brain connections and interactions [2] and musical training brings about both structural and functional changes within various brain areas [1], [3]. More recently, functional changes related to musical practice were observed in the hippocampus [8]. Our present research has highlighted a similar structural brain difference in the hippocampus, a brain area that is classically dedicated to memory. Considered alongside our fMRI results, this newly-observed structural difference supports the idea that musical practice induces gray matter plasticity in the hippocampus.

### Conclusion

For the first time, our study has identified functional differences between musicians and nonmusicians during a musical long-term memory task. This pattern of activation appears broadly to combine the neural networks involved in episodic and semantic memory [37]. These functional brain differences indicate that when musicians hear familiar melodies, more perceptual and contextual details come to mind, linking autobiographical episodic memories with subjective recollection. In addition, we found structural brain differences in the hippocampus, an area classically devoted to memory processes. Our findings support the idea that musical training may be associated with the development of specific memory abilities, that could contribute to a greater cognitive reserve, which could reduce age-related decline in memory [52].

## Materials and Methods

### Participants

Forty normal right-handed volunteers (age range: 20–35 years) took part in the study (Table 1). Half of them were musicians (10 women and 10 men) and the other half were nonmusicians (10 women and 10 men). Musicians (22.85±3.05 years; mean education level: 15.15±0.99) were recruited from the local music conservatory and other music schools. They played a variety of different instruments (violin, cello, guitar, flute, recorder, trumpet, clarinet and piano). On average, they had begun their musical training at the age of 7.55 years (±1.87 years) and had been playing music without interruption until the time of the study (number of years of practice: 15.3±3.67). They had learned music theory for a minimum of 7 years and none of them had absolute pitch.

Nonmusicians (24.55±3.80 years; mean education level: 16.35±2.03) were selected according to the following stringent criteria: (1) none had ever taken part in musical performances or received music lessons (except for basic music education at secondary school (1 hour/week)); (2) they had to be “common listeners” (i.e. not music lovers, who tend to listen to one specific type of music); and (3) they had to score normally on a test of pitch perception.

All participants had no history of neurological disorders and gave their written informed consent prior to taking part. The research protocol was approved by the regional ethics committee (Comité de Protection des Personnes Nord Ouest III).

In addition, before the fMRI scanning session, we tested both musicians and nonmusicians during a semantic memory task used in our previous study [22] to ensure that they had the same semantic memory skills. In this task, the subjects heard the beginning of a well-known tune (31 stimuli), and had to decide whether the second part matched (i.e. was the right end) or not. No performance difference was observed between groups during this semantic memory task. The mean accuracy of performance for the musical stimuli was 83.07 (±8.72) for nonmusicians and 86.9 (±7.79) for musicians (t(38) = −1.23, p>0.2).

### Task and stimuli

During the scanning session, all participants were tested on a familiarity task, in which they had to judge the level of familiarity of 60 excerpts of melodies on a 4-point scale. Participants were instructed to press the button under the middle finger of their left hand if they were sure that they never had heard the melody before (Fam1), the button under the index finger of their left hand if they were not sure whether they had heard it before or not (Fam2), the button under the index finger of their right hand if they knew having heard it several times (Fam3) and the button under the middle finger of their right hand if they knew it extremely well (Fam4). Participants were allowed to give their response when they want, from the beginning of the stimulus until the next stimulus (i.e. corresponding to approximately 8 sec). We did not ask them to access to the name of the melody or recalled personal memories to respond. Participants were instructed to close their eyes in order to focus better on the task. Melodies were purely instrumental tonal excerpts, taken from both the classical and modern repertoires (see Data S1 and Audio S1, for examples). All the melodies were selected from a pre-experimental study including 80 participants, both nonmusicians and musicians, matched with the present experimental sample. Thus, popular songs and melodies associated with lyrics were avoided in order to minimize verbal associations, as were those which might spontaneously evoke autobiographical memories, such as the “Wedding March” or melodies used in popular TV commercials. All the musical stimuli were played on a digital keyboard with a flute voice without orchestration and lasted between 4500 and 5500 milliseconds (see Data S1 and Audio S1). The interstimulus interval (ISI) varied between 2500 and 3500 milliseconds depending on the stimulus duration. We optimized the order of stimuli presentation using a Genetic Algorithm [53]. Order of presentation was fixed across the subjects. The stimuli were broadcast with an electrodynamic audio system ensuring an attenuation of scanner noise up to 45 dB (MR Confon headphone, Magdeburg Germany). The volume of the musical stimuli was adjusted individually to ensure that each subject could hear the stimuli clearly above the noise of the MRI scanner. Melodies were produced using the E-Prime software (Psychology Software Tools, Pittsburgh, PA) implemented within IFIS System Manager (Invivo, Orlando FL).

Following the scanning session, we performed a debriefing session in order to determine whether the melodies had evoked personal memories or mental imagery.

### Behavioral analyses

Using Statistica software, a two-way analysis of variance (ANOVA) was performed with the group as between-subjects factor with two modalities (nonmusicians and musicians), and the familiarity rating as a within-subject factor with four levels (Fam1, Fam2, Fam3, and Fam4). Similar analyses were conducted with the response time as within-subjects factor with the same four levels. If a significant interaction effect was observed these analyses were followed by Tukey's HSD *post hoc* tests.

### MRI scans

All images were acquired using the Philips (Eindhoven, The Netherlands) Achieva 3.0 T scanner. For each participant, a high-resolution T1-weighted anatomical image was first acquired using a 3D fast field echo sequence (3D-T1-FFE sagittal, TR  = 20 ms; TE  = 4.6 ms; flip angle  = 20°; 170 slices; slice thickness  = 1 mm; FOV  = 256×256 mm^2^; matrix  = 256×256; acquisition voxel size  = 1×1×1 mm^3^), followed by a high-resolution T2-weighted anatomical image (2D-T2-SE sagittal, SENSE factor  = 2; TR  = 5500 ms; TE  = 80 ms; flip angle  = 90°; 81 slices; slice thickness  = 2 mm; FOV  = 256×256 mm^2^; matrix  = 256×256; acquisition voxel size  = 2×1×1 mm^3^) and a non-EPI T2 star image (2D-T2 Star-FFE axial, SENSE factor  = 2; TR  = 3505 ms; TE  = 30 ms; flip angle  = 90°; 70 slices; slice thickness  = 2 mm; FOV  = 256×256 mm^2^; matrix  = 128×128; acquisition voxel size  = 2×2×2 mm^3^).

Functional data were acquired using an interleaved 2D T2 star EPI sequence designed to reduce geometrical distortions and magnetic susceptibility artefacts (2D-T2 Star-FFE-EPI axial, SENSE factor  = 2; TR  = 2382 ms; TE  = 30 ms; flip angle  = 80°; 44 slices; slice thickness  = 2.8 mm; matrix  = 80×80; FOV  = 224×224 mm^2^; acquisition voxel size  = 2.8×2.8×2.8 mm^3^; 207 volumes per run). The 414 functional volumes were collected during two functional sessions of 207 volumes each, where the first six volumes, of each session, were discarded to allow for equilibration effects.

### fMRI data processing

Data were analyzed using statistical parametric mapping software (SPM5; Wellcome Department of Cognitive Neurology, Institute of Neurology, London). During the pre-processing steps, after slice timing correction, images were realigned on the first volume of the first run. Coregistration of the EPI volumes onto the T1 image was then carried out in three steps: (1) the non-EPI T2 star volume was first coregistered onto the mean EPI image of the two runs, (2) the T2 image was then coregistered onto the coregistered non-EPI T2 star volume, and finally (3) the T1 volume was coregistered onto the coregistered T2 image. The mean EPI image was warped to roughly match the non-EPI T2 star volume using the methodology developed and validated by Villain et al. [54] to reduce geometric distortions. The warping parameters were applied to all the session's EPI volumes. The T1 image was then segmented/normalized using the SPM5 ‘Segment’ procedure [55] with the ICBM/MNI priors and the resulting normalization parameters were applied to the T1, to the warped EPI volumes. Finally, the normalized EPI images were smoothed at 8 mm FWHM. A high-pass filter was implemented using a cutoff period of 128 s to remove the low-frequency drift in the time series.

Individual analyses: a general linear model was applied to each participant and brain responses were modeled at each voxel. To investigate the effects of familiarity on the parametric modulation of the canonical hemodynamic response function (HRF), a parametric regressor modelling familiarity was constructed, based on the familiarity judgments for each melody excerpt. Familiarity was coded on the 4-point scale (1 totally unfamiliar to 4 extremely familiar). This analysis was performed for each subject, in order to identify those regions where activation was associated with an increase or decrease in familiarity rating. Functional data were analyzed until the subject responded (mean response time 5.46 sec) to highlight mainly the familiarity processes and to minimize the influence of retrieval of more specific information as recollection processes about the item or subvocal labeling that could occur after their response. Movement parameters were also included as covariates of no interest in the design matrix.

Group analyses: educational level was modeled as a covariate to control for the significant difference, at *p*<0.05, observed between musicians and nonmusicians, in favor of the latter. A conjunction analysis was first performed to identify the cerebral substrates that varied with musical familiarity in the musician and nonmusician groups. We used a conjunction analysis based on the proposed “valid conjunction inference with the minimum statistic” [56]. In this test, each comparison in the conjunction was individually significant, corresponding to the valid test for a “logical AND”. In order to identify brain regions associated with musical expertise during the familiarity task, we then performed this analysis comparing musicians and nonmusicians. A random effects analysis was conducted using a two-sample *t*-test. The musicians vs. nonmusicians comparison was masked exclusively with the nonmusicians' negative parametric modulation contrast, in order to identify those brain areas that were more strongly activated in musicians than in nonmusicians for the positive correlation between the BOLD signal and the familiarity rating. Similar analyses were performed for the nonmusicians vs. musicians comparison and for the negative correlation.

The resulting set of voxel values was thresholded at *p*<0.001 (uncorrected) and only activations involving clusters of more than 50 voxels were reported. This threshold was chosen in the light of empirical studies showing that it protects against false positives [57].

### Voxel-based morphometry analyses

To assess whether the differences in activation patterns between musicians and nonmusicians were related to structural differences, we also performed a VBM analysis. The T1-weighted MRI data were analyzed after being segmented and normalized during the fMRI data pre-processing, using SPM5 software. As fMRI and anatomical MRI data have different original spatial resolutions, differential smoothing was applied to obtain images of equivalent effective smoothness, and thus of identical resultant resolution [58], [59]. To this end, we used an isotropic Gaussian kernel of 8.4 mm FWHM for the aMRI data of 1×1×1 mm^3^ resolution, resulting in an effective smoothness identical to the fMRI data of 2.8×2.8×2.8 mm^3^ smoothed at 8 mm FWHM [60]. Structural group comparisons between musicians and nonmusicians were then conducted, using a two-sample *t*-test within ‘a functional mask’. This mask was created at the group level using the musicians vs. nonmusicians comparison and included only the voxels for which musicians elicited significantly greater activation than nonmusicians (thresholded at p<0.001, k≥50), considering that we want to examine the influence of musical expertise on structural plasticity strictly into the neural network involved in long-term memory for music. So, this mask corresponded to the regions showing significant functional differences between the two groups and represented in Figure 3. Although we did not correct for multiple comparisons, the use of an uncorrected threshold of *p*<0.001 limited the risk of false positives, as we restricted our structural investigation to a hypothesis-driven selection of regions (i.e. areas observed in the functional analysis), instead of a classic whole-brain exploratory analysis, as recommended by Whitwell [61]. To control for the significant difference (*p*<0.05) observed in educational level between musicians and nonmusicians, we modeled the educational level as a covariate in this analysis.

### Voxel based multimodal analyses

Finally, we analyzed fMRI data in relation to structural imaging information, in order to determine whether the activation highlighted in our fMRI investigation matched the structural differences observed between musicians and nonmusicians, that is, whether increased activity associated with familiarity was solely the consequence of higher gray matter density in these areas. This analysis was performed using BPM software (Biological Parametric Mapping, [24]) which allowed us to conduct a voxelwise analysis of data obtained for the different modalities.

## Supporting Information

Audio S1 Examples of stimuli used during the familiarity task.
(0.38 MB MP3)

Data S1 List of the musical excerpts used in the musical familiarity task.
(0.07 MB DOC)
