# Supplementary material for: When Music and Long-Term Memory Interact: Effects of Musical Expertise on Functional and Structural Plasticity in the Hippocampus
Source: PLoS One. 2010 Oct 5;5(10):e13225. doi: 10.1371/journal.pone.0013225 (PMC2950159; doi:10.1371/journal.pone.0013225)
Supplement: Data S1 — List of the musical excerpts used in the musical familiarity task. (0.07 MB DOC) [file pone.0013225.s002.doc]

**List of the musical excerpts used in the musical familiarity task**

| **TITLE** | **COMPOSER** |  |
| --- | --- | --- |
| The persuaders | John Barry |  |
| Bonnie blue flag - Dixie | Daniel Decatur Emmet | |
| My name is nobody | Ennio Morricone | |
| The symphony No. 3 | Johannes Brahms | |
| Unknown 6 |  |  |
| Toccata and Fugue in D minor | Johann Sebastian Bach | |
| Looney Tunes | Carl Stalling |  |
| God Save the Queen | Jean-Baptiste Lully | |
| The Barber of Seville (excerpt one) | Gioacchino Antonio Rossini | |
| National Anthem of America | Francis Scott Key | |
| Unknown 5 |  |  |
| Oxygene Part one | Jean-Michel Jarre | |
| Unknown 8 |  |  |
| Concerto No. 1 in E major "Spring" Allegro 2 | Antonio Lucio Vivaldi | |
| Hungarian Rhapsody n°2 | Franz Liszt |  |
| Sabre Dance | Aram Khatchaturian | |
| Popcorn | Gershon Kingsley | |
| The Godfather | Nina Rota |  |
| Tannhauser Overture | Richard Wagner | |
| Unknown 1 |  |  |
| The Entertainer | Scott Joplin |  |
| The Magic Flute - Papageno air 2 | Wolfgang Amadeus Mozart | |
| The Galop | Jacques Offenbach | |
| Te deum, eurovision song | Marc-Antoine Charpentier | |
| Unknown 3 |  |  |
| Clarinet Concerto K,622 - adagio | Wolfgang Amadeus Mozart | |
| Little Flower | Sidney Bechet | |
| Unknown 4 |  |  |
| Trio no.2 in E | Franz Peter Schubert | |
| Ode to joy | Ludwig van Beethoven | |
| Little night music (excerpt one) | Wolfgang Amadeus Mozart | |
| The Barber of Seville (excerpt two) | Gioacchino Antonio Rossini | |
| Unknown 14 |  |  |
| The Colonel Bogey | Maurice Jarre | |
| Pictures at an exhibition - First Promenade | Modest Mussorgsky | |
| Theme tune of Hawaii Five-O television series | Morton Stevens | |
| Concerto No. 1 in E major "Spring" | Antonio Lucio Vivaldi | |
| Army of Sambre-et-Meuse | Robert Planquette | |
| Unknown 2 |  |  |
| Battle Hymn of the Republic | Julia Ward Howe | |
| Musical Moment | Franz Peter Schubert | |
| Kalinka | Ivan Petrovitch Larionov | |
| Danse macabre | Camille Saint-Saëns | |
| Unkwown 9 |  |  |
| Funeral march of a marionette | Charles-François Gounod | |
| Peer Gynt - Morning | Edvard Grieg |  |
| Funeral March Sonata No. 2 | Frédéric Chopin | |
| The fifth symphony | Ludwig van Beethoven | |
| Theme tune of French Child Tv Serie | Dominique Thiel | |
| Unknown 10 |  |  |
| Lullaby | Johannes Brahms | |
| Polovtsian Dances | Alexander Borodin | |
| Lawrence of Arabia | Maurice Jarre | |
| Jesus, Joy of Man's desiring | Johann Sebastian Bach | |
| Unknown 12 |  |  |
| Brandenburg concerto | Johann Sebastian Bach | |
| Minuet | Luidi Rodolfo Boccherini | |
| Little night music (excerpt two) | Wolfgang Amadeus Mozart | |
| Unknown 7 |  |  |
| Turkish March | Wolfgang Amadeus Mozart | |
